# Adaptive Environmental Source Localization and Tracking with Unknown Permittivity and Path Loss Coefficients †

**DOI:** 10.3390/s151229852

**Published:** 2015-12-10

**Authors:** Barış Fidan, Ilknur Umay

**Affiliations:** Department of Mechanical and Mechatronics Engineering, University of Waterloo, 200 University Ave W, Waterloo, ON N2L 3G1, Canada; iumay@uwaterloo.ca

**Keywords:** localization, sensor networks, path loss coefficient, RSS, TOF

## Abstract

Accurate signal-source and signal-reflector target localization tasks via mobile sensory units and wireless sensor networks (WSNs), including those for environmental monitoring via sensory UAVs, require precise knowledge of specific signal propagation properties of the environment, which are permittivity and path loss coefficients for the electromagnetic signal case. Thus, accurate estimation of these coefficients has significant importance for the accuracy of location estimates. In this paper, we propose a geometric cooperative technique to instantaneously estimate such coefficients, with details provided for received signal strength (RSS) and time-of-flight (TOF)-based range sensors. The proposed technique is integrated to a recursive least squares (RLS)-based adaptive localization scheme and an adaptive motion control law, to construct adaptive target localization and adaptive target tracking algorithms, respectively, that are robust to uncertainties in aforementioned environmental signal propagation coefficients. The efficiency of the proposed adaptive localization and tracking techniques are both mathematically analysed and verified via simulation experiments.

## 1. Introduction

There has been significant research interest in the use of mobile sensory units and wireless sensor networks (WSNs) in various application areas, including environmental monitoring, especially in the last two decades. Typical mobile sensory units for environmental monitoring are autonomous vehicles (AVs) with certain types of sensor loads, and typical environmental monitoring WSNs are coordinated teams of such AVs, as well as sensor arrays on individual AVs. The main tasks of the environmental monitoring AVs and WSNs are localizing and state-observing various target objects, including animals, fire sources, fire fighter units, radioactive and biochemical emission sources and electromagnetic signal sources [1,2,3,4]. A key component in AV-based environmental monitoring is sensor instrumentation and localization algorithms utilizing these sensors. For localization, sensors are often used in sensor array or WSN forms. Use of such WSNs on AVs, e.g., UAVs, have various environmental applications, such as motion tracking, precision agriculture, coastline monitoring, rescue tasks, detecting and tracking fire, chemical and radioactive sources and pollutants [3,4,5,6,7,8,9,10,11,12].

Accurate signal-source and signal-reflector target localization via the aforementioned sensory units and WSNs requires precise knowledge of specific signal propagation properties of the environment. Such properties can be modelled by certain diffusion or propagation formulas, which involve some environmental coefficients, which are specific to the particular setting and which may be constant or time/space dependent. Environmental coefficients for radiation tracking and fire positioning are, respectively, radioactive sensor detection count rate and fire propagation velocity [6,8,9]. For electromagnetic signal source or reflector localization, typically, received signal strength (RSS) and time-of-flight (TOF)-based range sensors are used. Modelling of electromagnetic signal propagation for use by such sensors is more advanced [1,2]. The corresponding environmental coefficients are the path loss coefficient (*η*) for RSS and the signal permittivity coefficient (*ε*) for TOF.

In the literature, some preliminary studies for position tracking of radioactive and fire sources based on environmental coefficients are introduced in [6,8,9]. However, these studies either provide some rough data or assume *a priori* data from measurements on the environmental coefficients, which are the count rate for a radioactive source and the velocity of the hot gasses for fire localization. Electromagnetic signal source localization has various environmental monitoring applications, including surveillance of environmental (UAV, fire-fighter, robot) assist units, surveillance of objects tagged by electromagnetic signal sources or reflectors, surveillance of environmental intruders and positioning for rescue tasks [4,5,13,14]. A particular application is in fire-rescue systems, aiming at recognition and localization of the fire fighters [13,14].

Since localization algorithms are vulnerable to inaccuracies in the knowledge of the environmental coefficients, various approaches are proposed in the literature for the estimation of these coefficient or the compensation of uncertainties in the algorithms [15,16,17,18,19]. These approaches in general have a recursive nature and either still carry a significant amount of inaccuracy or require significant computational complexity for training and iteration of the estimation algorithms.

In this paper, we propose a more direct and static calculation technique for estimating the environmental coefficients, the path loss coefficient (*η*) for RSS and the signal permittivity coefficient (*ε*) for TOF, using a range sensor triplet during adaptive localization and tracking of a signal source by a mobile agent equipped with this sensor triplet. The triplet is designed to have a fixed rigid geometry where the *z*-coordinates of the sensors are equidistant. The proposed environmental coefficient estimation technique is integrated with a recursive least squares (RLS)-based adaptive localization scheme and an adaptive motion control law, to construct adaptive target localization and adaptive target tracking algorithms, respectively, that are robust to uncertainties in the aforementioned environmental signal propagation coefficients. The efficiency of the proposed adaptive localization and tracking techniques is both mathematically analysed and verified via simulation experiments.

Although the focus of this paper is on the localization of electromagnetic signal sources and reflectors in the environment and monitoring of objects based on such localization, the techniques studied in the paper have potential to be applied to the localization of the aforementioned fire source, radioactive emission source or biochemical source applications, as well.

The rest of the paper is organized as follows: The target localization and tracking problems of interest are defined, and the TOF and RSS-based range measurement and localization methods are briefly explained in Section 2. The details of the proposed environmental coefficient estimation technique are provided in Section 3. Section 4 and Section 5 present, respectively, the adaptive localization and adaptive tracking control designs. Simulation test results are provided in Section 6. The paper is concluded with some final discussions and remarks provided in Section 7.

## 2. Distance-Based Localization and Tracking

In this section, we formally state the source localization and tracking problems of interest and present the considered sensor instrumentation setting. The main principles and mathematical modelling of RSS and TOF-based distance measurement techniques are presented in Section 2.2 and Section 2.3. The effect of the environment on these techniques is briefly discussed in Section 2.4. For the methodology, we propose later to overcome the environmental uncertainties, use of a single sensor unit on the UAV is not sufficient; a sensor triplet, as a minimal sensor array, is required to be used. Hence, the problem definition in the next subsection assumes the use of a sensor triplet.

### 2.1. Localization and Tracking Problems

Consider a moving UAV equipped with sensor triplet $S=(S_{1},S_{2},S_{3})$, where the sensors are identical and sense the intensity of the signal emitted by a target source located at some unknown position

(1) $$p_{T}={\left[x_{T},y_{T},z_{T}\right]}^{T}$$

Note that $p_{T}$ may be time varying. Denote the position of the UAV at time instant $t=kT_{s}$ for k=0,1,2,⋯, where $T_{s}$ is the common sampling time used by the UAV sensors and processors, by

(2) $$p\left[k\right]={\left[x[k],y[k],z[k]\right]}^{T}$$

and the position vector of each sensor $S_{i}$ by

(3) $$p_{i}\left[k\right]={\left[x_{i}\left[k\right],y_{i}\left[k\right],z_{i}\left[k\right]\right]}^{T}$$

Assume that $p_{i}\left[k\right]$ and the target-sensor distance

(4) $$d_{i}\left[k\right]=∥p_{T}-p_{i}\left[k\right]∥$$

for each sensor $S_{i}$ are available to the processing unit of the sensory UAV. For simplicity, let the UAV position (body reference point) be defined as that of $S_{2}$, *i.e.*, let

(5) $$p\left[k\right]=p_{2}\left[k\right]$$

and hence, the target-UAV distance is defined as

(6) $$d\left[k\right]=∥p_{T}-p\left[k\right]∥$$

**Figure 1:**
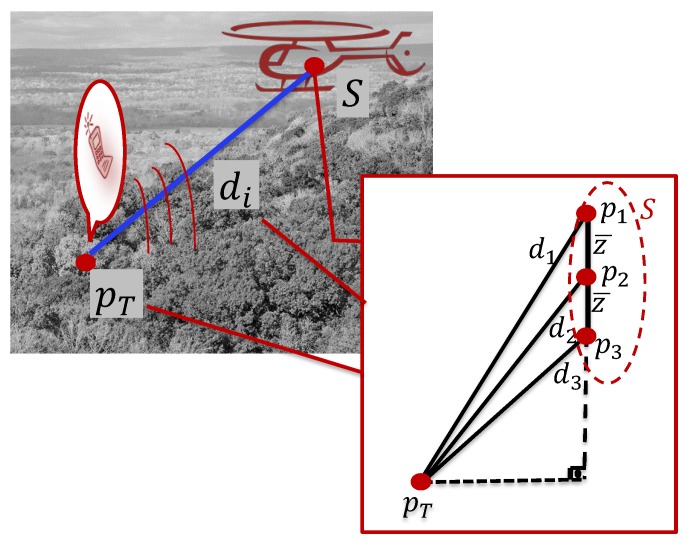
An illustration of the localization task setting and the proposed sensor array geometry, mobile sensor triplet unit (MSTU).

The 3D Localization Problem is to generate on-line estimate ${\hat{p}}_{T}\left[k\right]$ of $p_{T}$ using the measurements of $d_{i}\left[k\right]$ and $p_{i}\left[k\right]$. An illustration of the localization task setting is given in Figure 1.

In many practical cases, the UAV altitude with respect to the target *T*, e.g., when *T* is a ground target, is maintained constant and/or available for measurement, and the practical localization is to find the *x* and *y* coordinates of *T*. Accordingly, the (reduced order) Lateral Localization Problem is to generate on-line estimate ${\hat{p}}_{TL}\left[k\right]$ of $p_{TL}={\left[x_{T},y_{T}\right]}^{T}$ using the measurements of $d_{i}\left[k\right]$ and $p_{i}\left[k\right]$ and knowledge of $z_{T}$.

The Lateral Tracking Problem is to produce the control input for the UAV, using $d_{i}$ and $p_{i}$ measurements, such that $p_{L}\left[k\right]={\left[x[k],y[k]\right]}^{T}$ asymptotically converges to $p_{TL}$. For brevity, we skip the low level dynamic control design, assume perfect tracking of a velocity command and focus on the generation of the lateral velocity input

(7) $$v_{L}\left[k\right]={\dot{p}}_{L}\left[k\right]={\left.\frac{dp_{L}}{dt}\right|}_{t=kT_{s}}$$

as the high level kinematic control input only.

Note that, in both of the Localization and Lateral Tracking Problems defined above, $p_{T}$ may be time varying. Even though it is treated as constant in adaptive localization and tracking control scheme designs, simulation scenarios with time-varying $p_{T}$ are successfully tested, as demonstrated in Section 6.

### 2.2. RSS-Based Techniques

RSS is a distance measurement technique based on the signal power (or strength) measured by a receiver located at the sensor [1,20]. In a generic RSS setting, the target signal source, which is required to be localized, emits a signal with original power $P_{T}$. The power $P_{S}$ received by *S* follows an exponential decay model, which is a function of $P_{T}$, the distance $d_{T}$ between *S* and *T* and a coefficient *η* modelling the signal propagation behaviour in the corresponding environment, called the path loss coefficient (exponent). The widely-used corresponding mathematical model is

(8) $$P_{S}=K_{l}P_{T}d_{T}^{-\eta }$$

where $K_{l}$ represents other factors that include the effects of antenna height and antenna gain. $K_{l}$ is often considered to be log-normal and is often ignored in algorithm design leading to the simplified model

(9) $$P_{S}=P_{T}d_{T}^{-\eta }$$

The RSS technique often provides cost savings over deploying localization-specific hardware, and all current standard radio technologies, such as Wi-Fi and ZigBee, provide RSS measurements. However, RSS can have multi-path effects that include shadowing, reflection, diffraction and refraction due to unpredictable environmental conditions, particularly for indoor applications [21]. In modelling, these effects are also lumped and included in the coefficient $K_{l}$ of Equation (8).

### 2.3. TOF-Based Techniques

In TOF-based techniques, each sensor is composed of a transmitter unit, a receiver unit and a precision timer. The transmitter emits a signal, which is reflected by the target *T* and received by the receiver; and the time of flight, *i.e.*, the time elapsed between the signal’s emission and receiving of its reflection, is used to deduce the distance between the sensor and the target *T*. The environmental characteristics are summarized in the electromagnetic (e.g., radio-frequency (RF)) signal propagation velocity

(10) $$v=\frac{c}{\sqrt{\varepsilon }}$$

where *c* is the speed of light and *ε* denotes the (relative) permittivity coefficient.

Range is calculated by multiplying this propagation velocity and the measured TOF value. The corresponding mathematical model [22] can be formulated as

(11) $$t_{F}=\frac{2d_{T}}{v_{ave}}=d_{T}\sqrt{\bar{\varepsilon }}$$

where:

$$\bar{\varepsilon }=\frac{4\varepsilon }{c^{2}}=\frac{4}{v_{ave}^{2}}$$

Here, it is assumed that the TOF sensor emits a signal at $t_{D}=kT_{S}$ with a sampling period $T_{S}>0$ and stores the TOF $t_{F}$ value when the signal is received back at time $t\left[k\right]=kT_{S}+t_{F}\left[k\right]$. $T_{S}$ is chosen large enough to enable TOF measurements to satisfy $t_{F}\left[k\right]\ll T_{S}$ for any *k*.

The value of the TOF $t_{F}$ above can be measured using the phase of the received narrow-band carrier signal or via direct measurement of the arrival time of a wide-band narrow pulse [23]. The TOF-based technique, in general, requires strict time synchronization for the target and the receiver(s) [1].

### 2.4. Effect of the Environment

Information about the path loss exponent *η* for RSS-based techniques and the relative permittivity *ε* for TOF-based techniques have a vital effect on the measurement [15]. In many practical settings, these parameters are unknown and even variable in some due to the influences of variances on the weather conditions, human behaviour and the actuator effect at the anchor nodes. It is shown that using the wrong data on the path loss coefficient, *η*, has a huge effect on the accuracy of the position estimate [18].

Finding accurate estimation of these parameters is studied in the literature [16,17,18,19]. Most of the relevant works follow recursive algorithms involving training by data off-line or two-step on-line coefficient estimation and localization based on the estimate coefficients [16,17]. The off-line identification approaches require a large amount of training data for producing accurate estimates of the coefficients. The two-step on-line iterative approaches, on the other hand, may not lead to a successful level of accuracy during the joint coefficient estimation and localization process. The work in [15,18], for example, proposes iterative methodologies for the RSS case to obtain the unknown target location and path loss coefficient of the environment simultaneously in 2D with lower complexity. The iteration in these methodologies has the iterative steps of estimating the position of the target for the latest estimate of the path loss coefficient, calculating the corresponding RSS estimate and RSS estimate error and the application of an LS-based search for iterating the path loss coefficient estimate in order to minimize the RSS estimate error. During this process, upper and lower bounds for the path loss coefficient are assumed to be known.

A more systematic RLS procedure to simultaneously estimate the target position and the environmental coefficient is proposed in [22], for a TOF setting. In this work, a linear parametric formulation of the estimation problem, having a separate lumped parameter vector for both the unknown position and the unknown environmental coefficient (average signal propagation velocity during TOF), is derived, and an RLS algorithm is designed for this formation. The RLS algorithm proposed in [22] is an automatic recursive algorithm not requiring any heuristic search, has guaranteed convergence properties and has tunable design coefficients for tuning transient performance trade-off between faster convergence and reduced sensitivity to measurement noise, as superior properties compared to [15,18]. Yet, since it solves the same essential simultaneous minimization problem, there is no loss of estimation accuracy.

In an attempt to separate the target position estimation and environmental coefficient estimation problems and to advance the estimation accuracy level, henceforth, we propose a geometric sensor array technique for the environmental coefficient estimation problem in this paper. In Section 3, we present this technique, which overcomes the aforementioned issues via static or instantaneous calculation based on certain geometric relations. The required additional cost is the use of triplets of sensors at the nodes of the WSN or the sensory mobile agent of interest in place of single sensor units. We later provide comparative simulations in Section 6, to demonstrate the performance of the methodology, compared to that of [22], noting the relation with the works [15,18] mentioned above.

## 3. The Coefficient Estimation Technique

Consider the 3D and Lateral Localization Problems defined in Section 2.1. These problems are defined assuming the availability of distance measurements $d_{i}\left[k\right]$, bypassing how $d_{i}\left[k\right]$ are produced processing the actual measurements of RSS or TOF by the sensor triplet $S=(S_{1},S_{2},S_{3})$. In this section, we present our proposed geometric technique to produce the estimates of $d_{i}\left[k\right]$ using the available RSS or TOF measurements, which is equivalent to estimation of the path loss coefficient *η* for RSS or the signal permittivity coefficient *ε*, noting the model Equations (8)–(11).

In our design, we assert the geometric formation of the sensor triplet *S* to be maintained as rigid, such that $S_{1}$, $S_{2}$ and $S_{3}$ are aligned in the *z* direction with constant spacing $\bar{z}$, as depicted in Figure 1. That is, let the position of $S_{i}$ at step *k* for i=1,2,3 be given by

(12) $$p_{i}\left[k\right]={\left[x\left[k\right],y\left[k\right],z_{i}\left[k\right]\right]}^{T}$$

where $z_{1}\left[k\right]=z\left[k\right]+\bar{z}$, $z_{2}\left[k\right]=z\left[k\right]$ and $z_{3}\left[k\right]=z\left[k\right]-\bar{z}$ for some constant $\bar{z}$. Note that the spacing $\bar{z}$ is known, since it is a design constant.

At each step *k*, note that

(13) $$d_{1}^{2}-d_{2}^{2}={\left(z+\bar{z}-z_{T}\right)}^{2}-{\left(z-z_{T}\right)}^{2}={\bar{z}}^{2}+2\bar{z}\left(z-z_{T}\right)$$

(14) $$d_{3}^{2}-d_{2}^{2}={\left(z-\bar{z}-z_{T}\right)}^{2}-{\left(z-z_{T}\right)}^{2}={\bar{z}}^{2}-2\bar{z}\left(z-z_{T}\right)$$

Adding Equations (13) and (14), we obtain

(15) $$d_{1}^{2}\left[k\right]-2d_{2}^{2}\left[k\right]+d_{3}^{2}\left[k\right]=2{\bar{z}}^{2}$$

We propose the use of Equation (15) for the estimation of the environmental coefficient, η[k] for RSS or $\bar{\varepsilon }\left[k\right]$ for TOF. The time dependence of these coefficients comes mainly from time variations in the position of *S* (and $p_{T}$ if the target is not stationary) and, hence, the time variation in the environment between *T* and *S*.

More specifically, in the case of TOF, using Equation (11), Equation (15) can be rewritten as

(16) $$\frac{t_{F1}^{2}\left[k\right]}{\bar{\varepsilon }\left[k\right]}-2\frac{t_{F2}^{2}\left[k\right]}{\bar{\varepsilon }\left[k\right]}+\frac{t_{F3}^{2}\left[k\right]}{\bar{\varepsilon }\left[k\right]}=2{\bar{z}}^{2}$$

and, hence

(17) $$\bar{\varepsilon }\left[k\right]=\frac{t_{F1}^{2}\left[k\right]-2t_{F2}^{2}\left[k\right]+t_{F3}^{2}\left[k\right]}{2{\bar{z}}^{2}}$$

Similarly, in the case of RSS, using Equation (9), for each sensor $S_{i}$ we have

(18) $$\frac{P_{T}}{P_{Si}}=d_{i}^{\eta }$$

where $P_{Si}$ denotes the signal power received by $S_{i}$. Hence, Equation (15) can be rewritten as

(19) $$f\left(\bar{\eta }\right)=\zeta_{1}^{\bar{\eta }}-2\zeta_{2}^{\bar{\eta }}+\zeta_{3}^{\bar{\eta }}=2{\bar{z}}^{2}$$

where $\zeta_{i}=\frac{P_{T}}{P_{si}}$ and $\bar{\eta }=\frac{2}{\eta }$.

In the RSS case, although we cannot obtain a closed form solution for the coefficient *η* (or $\bar{\eta }$) similar to Equation (17), pre-calculated look-up tables for Equation (19) can be used (if preferred, together with some iterative accuracy fine-tuning methods) to solve Equation (19) for $\bar{\eta }$. A detailed formal study of such a design is out of the scope of this paper. However, for an *ad hoc* solution, one can observe that, in Equation (19), $\zeta_{1}$, $\zeta_{2}$, $\zeta_{3}$, $2{\bar{z}}^{2}$ are known/measured numbers, and $\bar{\eta }$ is the only unknown. For a UAV tracking a ground target, we have $P_{S1}<P_{S2}<P_{S3}<P_{T}$, since $d_{1}>d_{2}>d_{3}$. Hence, we have $\zeta_{1}>\zeta_{2}>\zeta_{3}$. Further, typically, 2≤η≤5. Therefore, $0.4\leq \bar{\eta }\leq 1$. For typical settings, $f(\bar{\eta })$ in Equation (19) is monotonic with no local minimum in the interval $0.4\leq \bar{\eta }\leq 1$. Applying a three-step grid search, with six equal intervals of a size of 0.1 in the first step and 10 equal intervals of sizes 0.01 and 0.001 in the second and third steps, respectively, $\bar{\eta }$ can be calculated with an error tolerance of ±0.001. Such search is real-time implementable, and better results can be obtained using more steps.

## 4. Adaptive Source Localization Scheme

In this section, an RLS-based adaptive source localization scheme is designed to perform the target localization tasks of the 3D Localization Problem and the Lateral Localization Problem defined in Section 2.1. The adaptive localization scheme is to generate the estimate ${\hat{p}}_{T}\left[k\right]$ of $p_{T}$ using the information of $p_{i}\left[k\right]$, which is obtained by the self-positioning system of the UAV together with the geometric relation Equation (12) and $d_{i}\left[k\right]$, which is obtained using the proposed technique in Section 3. Similarly to [22], the unknown target position vector, ${\hat{p}}_{T}$, is treated as constant in the design, and the influence of the drifting of the target is analysed later. The adaptive localization scheme is designed as an RLS algorithm with a forgetting factor [22,24] based on a linear parametric model, separately derived for each of the 3D Localization and Lateral Localization Problems, in the sequel.

### 4.1. 3D Localization

We first study solution of the 3D Localization Problem. To derive a linear parametric model for this problem, from Equations (4) and (5), we have

(20) $$\begin{array}{ccc} d^{2}\left[k\right] & = & {\left(p\left[k\right]-p_{T}\right)}^{T}\left(p\left[k\right]-p_{T}\right)\\ & = & {∥p\left[k\right]∥}^{2}+{∥p_{T}∥}^{2}-2p_{T}^{T}p\left[k\right] \end{array}$$

Evaluating Equation (20) at steps *k* and k-1 and taking the difference, we obtain

(21) $$d^{2}\left[k\right]-d^{2}\left[k-1\right]={∥p\left[k\right]∥}^{2}-{∥p\left[k-1\right]∥}^{2}-2p_{T}^{T}\left(p\left[k\right]-p\left[k-1\right]\right)$$

which can be written in the linear parametric model form

(22) $$\begin{array}{ccc} \zeta [k] & = & p_{T}^{T}\phi \left[k\right] \end{array}$$

where ϕ[k] and ζ[k] are defined as

(23) $$\begin{array}{ccc} \phi [k] & = & p[k]-p[k-1] \end{array}$$

(24) $$\begin{array}{ccc} \zeta [k] & = & \frac{1}{2}\left({∥p\left[k\right]∥}^{2}-{∥p\left[k-1\right]∥}^{2}-\left(d^{2}\left[k\right]-d^{2}\left[k-1\right]\right)\right) \end{array}$$

Based on the linear parametric model Equations (22)–(24), various estimators can be designed to produce the estimate ${\hat{p}}_{T}$ of $p_{T}$. Next, we design an on-line RLS estimator based on the parametric model Equations (22)–(24). Following the design procedure in [24], we obtain the following RLS adaptive law with a forgetting factor and parameter projection:

(25) $$\begin{array}{ccc} {\hat{p}}_{T}\left[k\right] & = & Pr\left({\hat{p}}_{T}\left[k-1\right]+\Gamma \left[k\right]\phi \left[k\right]\epsilon \left[k\right]\right) \end{array}$$

(26) $$\begin{array}{ccc} \epsilon [k] & = & \zeta \left[k\right]-{\hat{p}}_{T}^{T}\left[k-1\right]\phi \left[k\right] \end{array}$$

(27) $$\begin{array}{ccc} \Gamma [k] & = & \frac{1}{\beta_{f}}\left(\Gamma \left[k-1\right]-\frac{\Gamma \left[k-1\right]\phi \left[k\right]\phi {\left[k\right]}^{T}\Gamma \left[k-1\right]}{\beta_{f}+\phi {\left[k\right]}^{T}\Gamma \left[k-1\right]\phi \left[k\right]}\right) \end{array}$$

where ϵ[k] is the (measurable) output estimate error, Γ[k] is the 3×3 dynamic adaptive gain matrix (called the covariance matrix), $0<\beta_{f}<1$ is the forgetting factor coefficient and Pr(.) is the parameter projection operator used to satisfy ${\hat{p}}_{T3}={\hat{z}}_{T}\in R_{z}$, where the target attitude is assumed to be known *a priori* to lie in the range $R_{z}=\left[z_{T,min},z_{T,max}\right]$. Initial covariance matrix $\Gamma \left[0\right]=\Gamma_{0}$ is selected to be positive definite, which guarantees together with Equation (27) that Γ[k] is positive definite for all *k*.

### 4.2. Lateral Localization

The Lateral Localization Problem is a relaxed form of the 3D Localization Problem, reducing its parametric model order by one. To derive the reduced order linear parametric model, we rewrite Equation (22) as

(28) $$\begin{array}{ccc} \zeta [k] & = & p_{T}^{T}\phi \left[k\right]\;=\;p_{TL}^{T}\phi_{L}\left[k\right]+z_{T}\phi_{z}\left[k\right] \end{array}$$

where $\phi_{L}\left[k\right]$ and $\phi_{z}\left[k\right]$ are defined as

(29) $$\begin{array}{ccc} \phi_{L}\left[k\right] & = & p_{L}\left[k\right]-p_{L}\left[k-1\right] \end{array}$$

(30) $$\begin{array}{ccc} \phi_{z}\left[k\right] & = & z[k]-z[k-1] \end{array}$$

Using the available information of $\phi_{z}\left[k\right]$, we obtain the reduced order linear parametric model

(31) $$\begin{array}{ccc} \zeta_{L}\left[k\right] & = & p_{TL}^{T}\phi_{L}\left[k\right] \end{array}$$

where $\zeta_{L}\left[k\right]$ is defined as

(32) $$\begin{array}{ccc} \zeta_{L}\left[k\right] & = & \frac{1}{2}\left({∥p\left[k\right]∥}^{2}-{∥p\left[k-1\right]∥}^{2}-\left(d^{2}\left[k\right]-d^{2}\left[k-1\right]\right)\right)-z_{T}\left(z\left[k\right]-z\left[k-1\right]\right) \end{array}$$

In the design of on-line RLS estimator for the reduced order parametric model Equation (31), we do not need the parameter projection for the *z*-coordinate any more. Further, the model order is two instead of three. Hence, the RLS adaptive law for this case is redesigned as follows:

(33) $$\begin{array}{ccc} {\hat{p}}_{TL}\left[k\right] & = & {\hat{p}}_{TL}\left[k-1\right]+\Gamma \left[k\right]\phi_{L}\left[k\right]\epsilon_{L}\left[k\right] \end{array}$$

(34) $$\begin{array}{ccc} \epsilon_{L}\left[k\right] & = & \zeta_{L}\left[k\right]-{\hat{p}}_{TL}^{T}\left[k-1\right]\phi_{L}\left[k\right] \end{array}$$

(35) $$\begin{array}{ccc} \Gamma_{L}\left[k\right] & = & \frac{1}{\beta_{f}}\left(\Gamma_{L}\left[k-1\right]-\frac{\Gamma_{L}\left[k-1\right]\phi_{L}\left[k\right]\phi_{L}{\left[k\right]}^{T}\Gamma_{L}\left[k-1\right]}{\beta_{f}+\phi_{L}{\left[k\right]}^{T}\Gamma_{L}\left[k-1\right]\phi_{L}\left[k\right]}\right) \end{array}$$

where $0<\beta_{f}<1$ is the forgetting factor coefficient, as before, and the adaptive gain (covariance) matrices $\Gamma_{L}\left[0\right]=\Gamma_{L0}$ and, hence, $\Gamma_{L}\left[k\right]$, for all k>0 are 2×2 and positive definite.

### 4.3. Stability and Convergence of the Adaptive Localization Laws

The adaptive localization law Equations (25) and (33) are discrete-time RLS algorithms with a forgetting factor (and parameter projection). Such algorithms are studied in detail in [24]. It is also established there and in the references therein that, for 3D localization, if ϕ[k] is persistently exciting (PE), *viz*., if it satisfies

(36) $$\lim_{K\to \infty }\lambda_{min}\left(\sum_{k=0}^{K}\phi \left[k\right]\phi^{T}\left[k\right]\right)=\infty$$

or if the 3×3 matrix

(37) $$\sum_{k=k_{0}}^{k_{0}+l-1}\phi \left[k\right]\phi^{T}\left[k\right]-\alpha_{0}lI$$

where *I* is the identity matrix and $\lambda_{min}\left(\cdot \right)$ denotes the minimum eigenvalue, is positive semi-definite for some $\alpha_{0}>0$, l≥1 and for all $k_{0}\geq 1$, then ${\hat{p}}_{T}\left[k\right]\to p_{T}$ as k→∞. The geometric interpretation of the above PE condition is that the UAV is required to avoid converging to planar motion, *i.e.*, to avoid p[k] converging to a certain fixed 2D plane.

Similarly, for 2D localization, if $\phi_{L}\left[k\right]$ is PE, then ${\hat{p}}_{TL}\left[k\right]\to p_{TL}$ as k→∞; with the geometric interpretation that the UAV is required to avoid converging to linear motion, *i.e.*, to avoid $p_{L}\left[k\right]$ converging to a certain fixed 1D line.

## 5. Adaptive Tracking Control

In this section, our proposed control scheme for the Lateral Tracking Problem defined in Section 2.1 is presented. Similarly to Section 4, the required information of $p_{i}\left[k\right]$ and $d_{i}\left[k\right]$ is obtained on-line using the self-positioning system of the UAV together with the geometric relation Equation (12) and the proposed environmental coefficient estimation technique in Section 3, respectively. The adaptive tracking control scheme is designed following a discrete-time version of the approach in [25].

**Figure 2:**
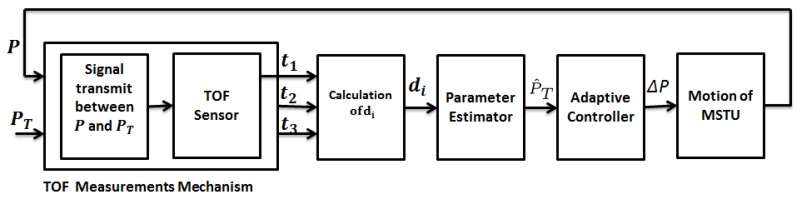
Block diagram of the proposed adaptive lateral target tracking control scheme.

The lateral tracking objective of Section 2.1 is considered as assigning a tracking control law to generate the lateral velocity $v_{L}\left[k\right]$ based on estimate ${\hat{p}}_{T}\left[k\right]$ of the unknown target position to achieve

(38) $$\lim_{k\to \infty }d_{L}\left[k\right]=0$$

where

(39) $$d_{L}\left[k\right]=∥p_{L}\left[k\right]-p_{LT}∥={\left(d^{2}\left[k\right]-{\left(z\left[k\right]-z_{T}\right)}^{2}\right)}^{1/2}$$

is available for measurement and, hence, can be used as a variable in the control law. In the design of the proposed adaptive target tracking control scheme, with the block diagram provided in Figure 2, we follow a certainty equivalence approach similar to [25], integrating three modular tools:

(i) The adaptive localization scheme of Section 4 to produce on-line estimate ${\hat{p}}_{T}\left[k\right]$ of target position $p_{T}$.
(ii) A motion control law fed by the estimate ${\hat{p}}_{T}\left[k\right]$ in place of unknown $p_{T}$ to generate the lateral velocity $v_{L}\left[k\right]$, aiming to drive the estimated lateral distance $∥p_{L}\left[k\right]-{\hat{p}}_{LT}\left[k\right]∥$ to zero.
(iii) A low amplitude periodic auxiliary control signal to be augmented to the motion control law to satisfy the PE condition needed for guaranteeing the convergence of the location estimate ${\hat{p}}_{T}\left[k\right]$ to $p_{T}$.

In the design of Modules (ii) and (iii), we adopt and discretize the continuous-time adaptive target pursuit design in [25] to form the following discrete (augmented) motion control law:

(40) $$v_{L}\left[k\right]=\frac{{\hat{p}}_{TL}\left[k\right]-{\hat{p}}_{TL}\left[k-1\right]}{T_{s}}-\beta_{c}\left(p_{L}\left[k\right]-{\hat{p}}_{TL}\left[k\right]\right)+f\left(d_{L}\left[k\right]\right)v_{a}\left[k\right]$$

to be applied to the motion dynamics, using zero order hold, as

(41) $${\dot{p}}_{L}\left(t\right)=v_{L}\left(t\right)=v_{L}\left[k\right],\;\text{for}\;kT_{s}\leq t<\left(k+1\right)T_{s}$$

where $\beta_{c}>0$ is the proportional control gain,

(42) $$v_{a}\left[k\right]=v_{a}\left(kT_{S}\right)=a_{\sigma }\left[\begin{array}{c} \sin a_{\sigma }kT_{s}\\ \cos a_{\sigma }kT_{s} \end{array}\right]$$

is the periodic auxiliary control signal with frequency $a_{\sigma }$, and f(·) is a strictly increasing and bounded function that is zero at zero and satisfies $f\left(d_{F}\right)\leq d_{F},\;\forall d_{F}>0$. The function f(·) is used to attenuate the auxiliary signal amplitude as the UAV gets closer to the target *T*.

Based on the analysis provided in [25], we observe the properties of $v_{a}\left(t\right)$ summarized in the following lemma:

**Lemma 1.**  *The auxiliary signal $v_{a}$ defined in Equation (42) satisfies the following:*

(*i*) *There exist positive $T_{1},\alpha_{i}>0$, such that for all t≥0, there holds:*
  $$\alpha_{1}∥v_{a}{\left(0\right)∥}^{2}I\leq \int_{t}^{t+T_{1}}v_{a}\left(\tau \right)v_{a}{\left(\tau \right)}^{⊤}d\tau \leq \alpha_{2}{∥v_{a}\left(0\right)∥}^{2}I.$$
(*ii*) *For every $\theta \in {ℜ}^{2}$, and every t>0, there exists $t_{1}\left(t,\theta \right)\in \left[t,t+T_{1}\right]$, such that $\theta^{⊤}v_{a}\left(t_{1}\left(t,\theta \right)\right)=0$.*
(*iii*) *For all t≥0, $∥v_{a}\left(t\right)∥=∥v_{a}\left(0\right)∥=a_{\sigma }$.*
(*iv*) *There exists a design constant $a_{\sigma }$, such that the discrete time signal $v_{a}\left[k\right]=v_{a}\left(kT_{s}\right)$ is PE.*

**Proof.**  (i) is a direct corollary of Lemma 8.1 of [26]. (ii) and (iii) are direct corollaries of, respectively, Theorem 5.1 and Lemma 3.1 of [25]. (iv) follows from (i) and (iii). □

Lemma 1 and classical arguments of the discretization of continuous-time dynamic systems lead to the validity of the boundedness and convergence results in Theorems 4.1 and 4.2 of [25] for our case, as well, as summarized in the following proposition:

**Proposition 1.**  Consider the closed-loop adaptive tracking control system composed of the adaptive law Equation (33), the motion control law Equation (40) and the motion dynamics Equation (41). Assume that $\beta_{c}>{\bar{\sigma }}^{\prime }$ for the upper bound ${\bar{\sigma }}^{\prime }$ defined in Lemma 1. Then, there exists a sufficiently small sampling time $T_{s}$, such that all of the closed-loop signals are bounded and $p_{L}\left[k\right]$ asymptotically converges to $p_{TL}$.

## 6. Simulations

In this section, we perform simulation testing of the proposed adaptive localization and target tracking schemes. First, we consider a scenario where the UAV is equipped with a TOF-based range sensor triplet. In all of the simulations, the actual average permittivity is taken to be $\varepsilon_{ave}=5$ considering solid objects and air humidity in the signal propagation paths for TOF sensors. The vertical spacing for the sensor triplet $S=(S_{1},S_{2},S_{3})$ is chosen as $\bar{z}=10$ cm, and the common sampling time is selected as $T_{s}=1$ s.

**Figure 3:**
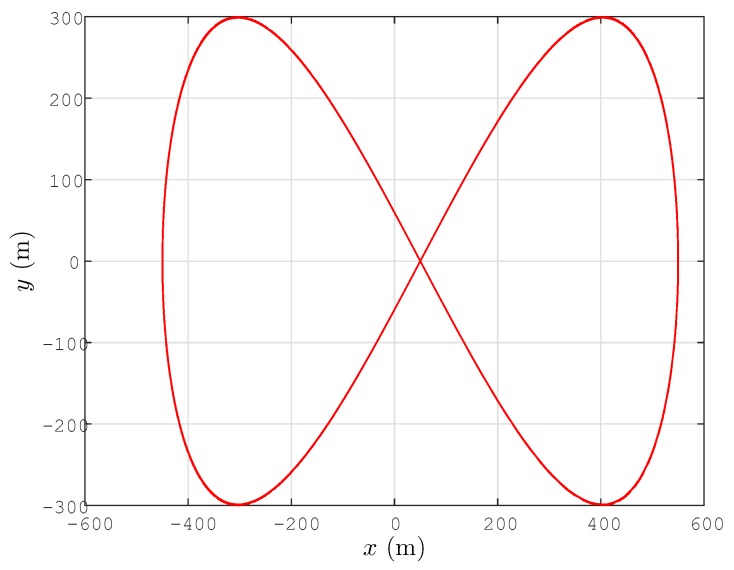
Lateral trajectory (x(t),y(t)) (m) (or (x[k],y[k])) of the UAV.

The task of the UAV is to estimate the location $p_{T}$ of (and track) a certain target *T*. For this task, the UAV uses the localization algorithm Equation (25), and in order to guarantee estimation convergence per the discussions at the end of Section 4, it follows a PE path, *i.e.*, a path satisfying *ϕ* to be PE. As such a PE path, we consider the following path, whose *x* and *y* coordinate components are plotted in Figure 3:

(43) $$\begin{array}{ccc} & & x(t)=500\cos(0.1t)+50\;m \end{array}$$

(44) $$\begin{array}{ccc} & & y(t)=300\cos(0.2t)\;m \end{array}$$

(45) $$\begin{array}{ccc} & & z(t)=5\sin(0.1t)+39\;m \end{array}$$

We consider the following design parameter selections for the localization algorithm Equation (25):

(46) $$\begin{array}{ccc} & & \beta_{f}=0.9 \end{array}$$

(47) $$\begin{array}{ccc} & & \Gamma [0]=I \end{array}$$

(48) $$\begin{array}{ccc} & & {\hat{p}}_{T}\left[0\right]={\left[0,0,0\right]}^{T}\;m \end{array}$$

We consider two cases for the target position $p_{T}$ in the following two subsections. As a continuation of the discussion at the end of Section 2.4, we compare the results using our prosed algorithm with the results using the simultaneous location and permittivity coefficient estimation scheme of [22] (for the same setting). As opposed to the proposed approach in Section 3, which gives very accurate results instantaneously, in the simulation of the scheme of [22], permittivity coefficient estimation is done recursively together with the target location estimation. The initial permittivity coefficient estimate for this recursive estimation is chosen as ${\hat{\varepsilon }}_{ave}\left[k\right]=10$.

### 6.1. Stationary Target Localization

First, we consider a stationary target located at

(49) $$\begin{array}{ccc} & & p_{T}={\left[100,75,8\right]}^{T}\;m \end{array}$$

**Figure 4:**
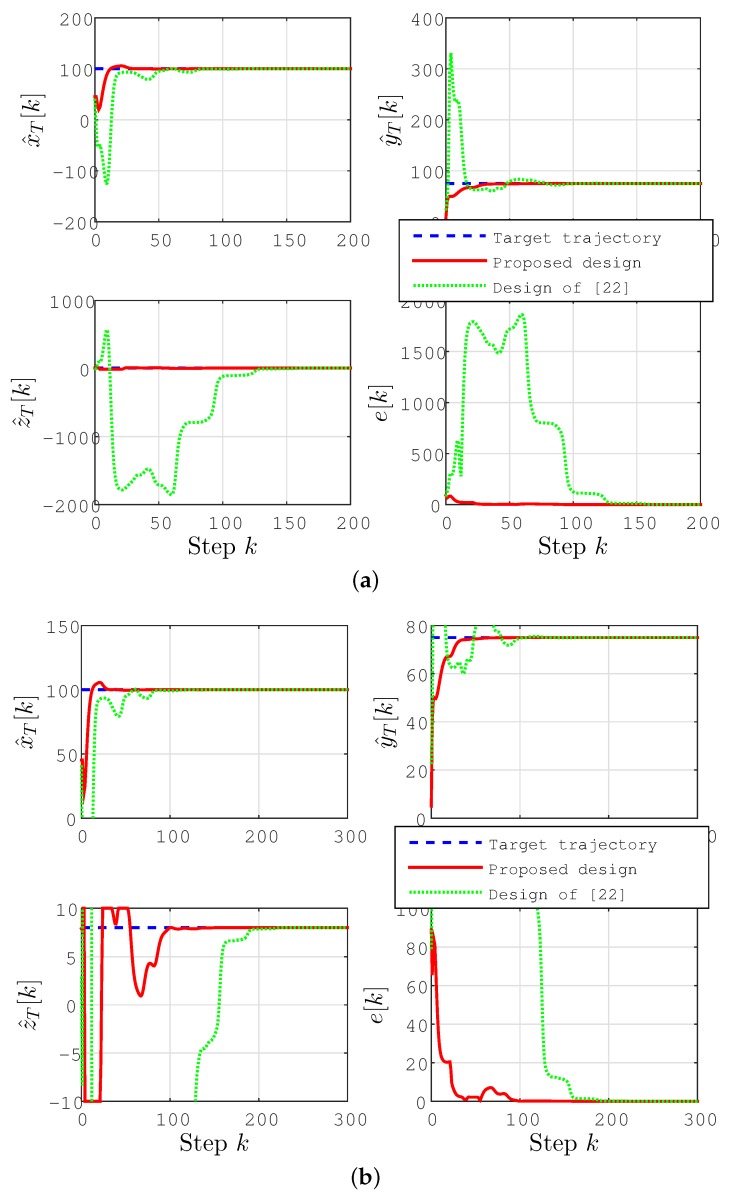
Location estimate ${\hat{p}}_{T}\left[k\right]$ and estimation error $e\left[k\right]=∥{\hat{p}}_{T}\left[k\right]-p_{T}∥$(m) for the stationary target case. (**b**) The scaled version of (**a**) to provide the details of the convergence characteristics for the proposed scheme.

**Figure 5:**
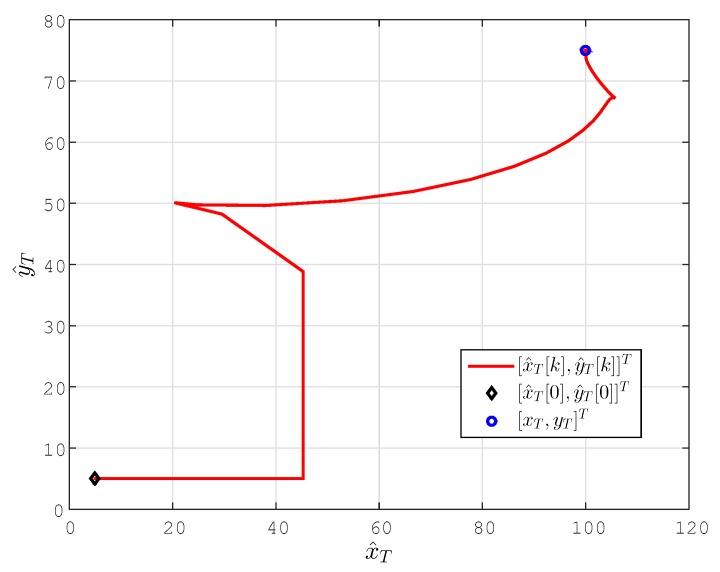
Lateral coordinate estimates $({\hat{x}}_{T}\left[k\right],{\hat{y}}_{T}\left[k\right])\;\left(m\right)$ for the stationary target case.

The localization results for this case are plotted in Figure 4 and Figure 5. In Figure 4, we compare the results using our prosed algorithm with the results using the simultaneous location and permittivity coefficient estimation scheme of [22] (for the same setting).

It is clearly seen in these figures that, using the prosed design, all of the coordinates of the position estimate ${\hat{p}}_{T}\left[k\right]$ rapidly converge to their actual values, leading the estimation error $e\left[k\right]=∥{\hat{p}}_{T}\left[k\right]-p_{T}∥$ to converge to zero. The estimates converge significantly faster than those using the design of [22], with significantly smaller overshoot/undershoot. One can further enhance the performance of localization by fine-tuning the design parameters given above.

### 6.2. Drifting Target Localization

As a second scenario, we consider a slowly drifting target *T* with position

$$\begin{array}{ccc} & & x_{T}\left(t\right)=0.1t+\left(2\sin\left(0.05t\right)+100\right)\;m\\ & & y_{T}\left(t\right)=0.05t+\left(2\sin\left(0.05t\right)+75\right)\;m\\ & & z_{T}\left(t\right)=\left(0.5\sin\left(0.01t\right)+8\right)\;m \end{array}$$

and hence, velocity

$$\begin{array}{ccc} V_{T} & = & \left[(0.1+0.1\cos(0.05t)),(0.05+0.1\cos(0.05t)\right)\\ & & {\left(0.005\cos\left(0.01t\right)\right)]}^{T}\;m/s \end{array}$$

**Figure 6:**
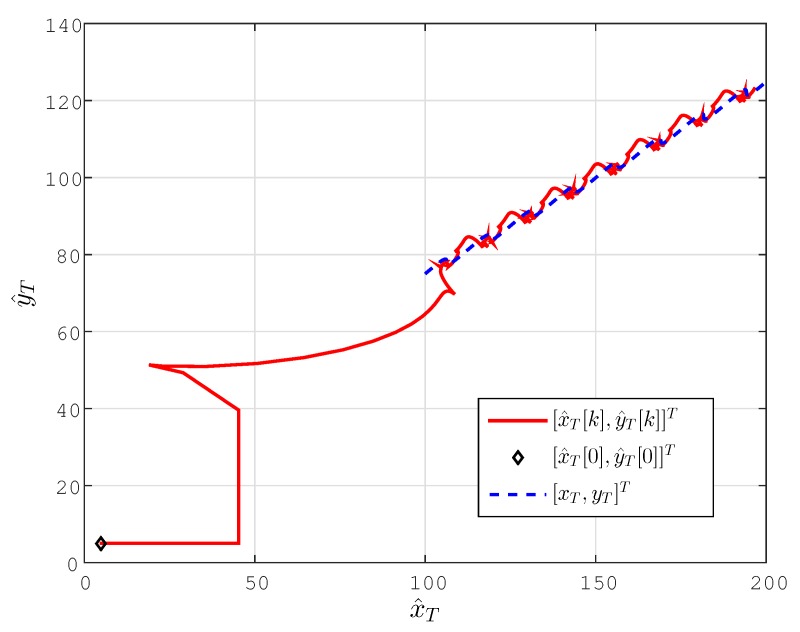
Lateral coordinate estimates $\left({\hat{x}}_{T}\left[k\right],{\hat{y}}_{T}\left[k\right]\right)$ (m) for the drifting target case.

**Figure 7:**
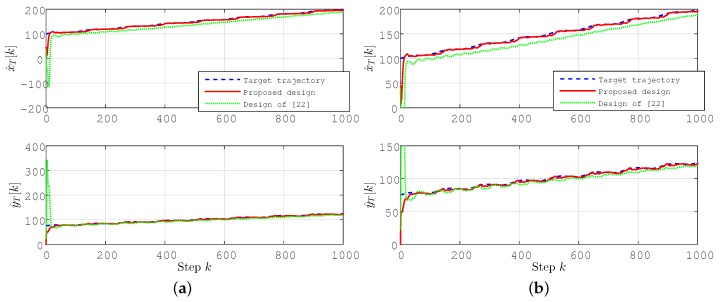
Location estimate ${\hat{p}}_{T}\left[k\right]$ for the drifting target case. (**b**) The scaled version of (**a**) to provide the details of the convergence characteristics for the proposed scheme.

The localization results for this case are plotted in Figure 6 and Figure 7.

As can be seen in these figures, convergence characteristics are comparable to the stationary target case; however, due to the motion of the target, perfect convergence is impossible as long as the velocity $v_{T}$ of the target is not known *a priori*. The estimation accuracy, however, is significantly better, and the estimates converge significantly faster than those using the design of [22], with significantly smaller overshoot/undershoot.

### 6.3. Drifting Target Tracking

**Figure 8:**
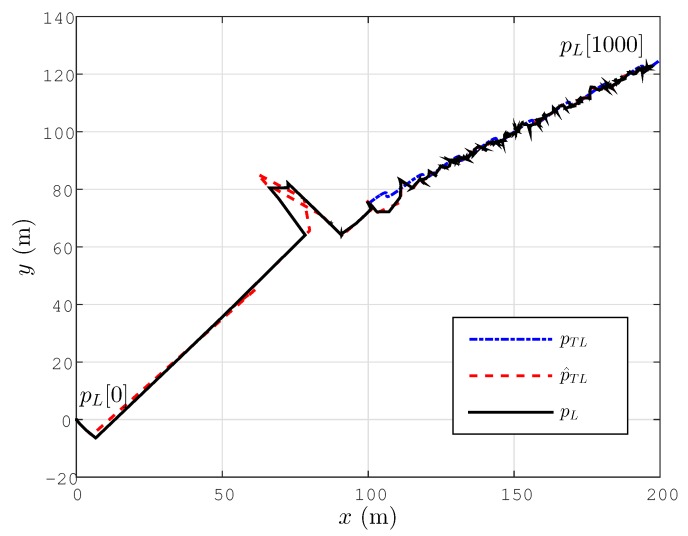
Lateral tracking control for a drifting target for the simplified motion dynamics model Equation (41).

Next, we consider the tracking problem for the scenario considered in the previous subsection. The target motion is the same. The motion control law designs are selected as $\beta_{c}=3$, $a_{\sigma }=0.01$. The simulation results are shown in Figure 8. We can easily see that the $p_{L}\left[k\right]$ values converge to ${\hat{p}}_{TL}\left[k\right]$, as well as $p_{TL}\left[k\right]$ values. The simulation results demonstrate that the tracking task of the target is achieved. Better tracking performance can be obtained by fine-tuning the adaptive localization and target tracking control design terms.

**Figure 9:**
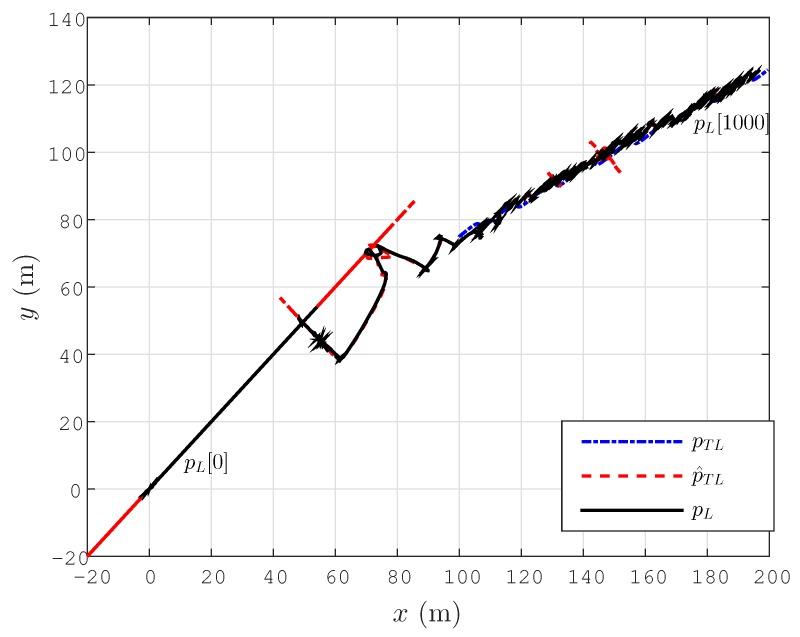
Lateral tracking control for a drifting target for the detailed motion dynamics model Equation (50).

To further examine the ignored actuator dynamics and disturbance effects, the simulation above is performed for the following modified version of the motion dynamics model Equation (41):

(50) $$\begin{array}{ccc} {\dot{p}}_{L} & = & \frac{1}{\tau_{a}s+1}\left[v_{L}\right]+w_{v} \end{array}$$

(51) $$\begin{array}{ccc} v_{L}\left(t\right) & = & v_{L}\left[k\right],\;\text{for}\;kT_{s}\leq t<\left(k+1\right)T_{s} \end{array}$$

where $\frac{1}{\tau_{a}s+1}$ is the transfer function of the actuator dynamics with time coefficient considered to be $\tau_{a}=0.2$ (s), and $w_{v}$ is a band limited white noise with power 0.1, representing further motion control disturbances. The simulation results shown in Figure 9 demonstrate that the results are comparable to those in Figure 8.

## 7. Conclusions

In this paper, a geometric cooperative technique has been proposed to instantaneously estimate permittivity and path loss coefficients in electromagnetic signal source and reflector localization and tracking tasks, focusing on environmental monitoring applications. The details of the technique are provided for RSS and TOF-based range sensor settings. The use and performance of the technique are analysed and demonstrated on its integration with a discrete time RLS-based adaptive localization and target tracking control schemes. A set of UAV-based target localization and tracking simulation scenarios are provided to demonstrate the effectiveness of the integration of the adaptive localization and tracking schemes and the proposed coefficient estimation technique. The proposed technique involves only static instantaneous calculation based on a certain geometric relation and, hence, provides a computationally efficient way to solve the localization problems in environments with unknown permittivity and path loss coefficients, compared to the other relevant techniques proposed in the literature.

Ongoing and future follow up research directions include more formal analysis of various localization and tracking schemes using the proposed coefficient estimation technique, applications in other domains, such as biomedical monitoring [27], and real-time implementation and experimental testing. Implementation and testing of such a system can be considered in two layers, the hardware layer and the software layer. For the hardware layer implementation, a sensor triplet unit, as described in Section 2.2 and Section 2.3, together with onboard CPU and communication (to broadcast the on-line localization/tracking information) units at the mobile agent, which can be mounted on the surveillance UAV. The software layer implementation will include the low level coding of the proposed algorithms, which are all real-time implementable, and further embedded software for the CPU interface with sensor and communication units. The setup for this architecture can be constructed using standard hardware units and software, such as those used for the experiments in [28,29].

## References

[1] Mao G., Fidan B. (2009). Localization Algorithms and Strategies for Wireless Sensor Networks.

[2] Yüce M.R., Khan J.Y. (2012). Wireless Body Area Networks Technology, Implementation and Applications.

[3] Lomax A.S., Corso W., Etro J.F. (2005). Employing unmanned aerial vehicles (UAVs) as an element of the Integrated Ocean Observing System. MTS/IEEE Proc..

[4] Susca S., Bullo F., Martinez S. (2008). Monitoring environmental boundaries with a robotic sensor network. IEEE Trans. Control Syst. Technol..

[5] Arnold T., de Biasio M., Fritz A., Leitner R. UAV-based multispectral environmental monitoring. Proceedings of the 2010 IEEE Sensors Conference.

[6] Wang S., Berentsen M., Kaiser T. (2005). Signal processing algorithms for fire localization using temperature sensor arrays. Fire Saf. J..

[7] Liu Z. A supervisory approach for hazardous chemical source localization. Proceedings of the 2013 IEEE International Conference on Mechatronics and Automation (ICMA).

[8] Howse J.W., Ticknor L.O., Muske K.R. (2011). Least squares estimation techniques for position tracking of radioactive sources. Automatica.

[9] Brennan S.M., Mielke A.M., Torney D.C., Maccabe A.B. (2004). Radiation detection with distributed sensor networks. IEEE Comput..

[10] Campbell M. (2012). Sensor Systems for Environmental Monitoring.

[11] Lin J., Xiao W., Lewis F.L., Xie L. (2009). Energy-efficient distributed adaptive multisensor scheduling for target tracking in wireless sensor networks. IEEE Trans. Instrum. Meas..

[12] Yoon S.H., Ye W., Heidemann J., Littlefield B., Shahabi C. (2011). SWATS: Wireless sensor networks for steam-flood and water-flood pipeline monitoring. IEEE Netw..

[13] Liu Y., Yuan X., Chen Y., Lin Y. (2011). Dynamic localization research for the fire rescue system. Procedia Eng..

[14] Ge Q., Wen C., Duan S. (2014). Fire localization based on range-range-range model for limited interior space. IEEE Trans. Instrum. Meas..

[15] Wang G., Chen H., Li Y., Jin M. (2012). On received-signal-strength based localization with unknown transmit power and path loss exponent. IEEE Wirel. Commun. Lett..

[16] Gezici S. (2008). A survey on wireless position estimation. Wirel. Pers. Commun..

[17] Çamlıca A., Fidan B., Yavuz M. Implant localization in the human body using adaptive least square based algorithm. Proceedings of the ASME 2013 International Mechanical Engineering Congress and Exposition.

[18] Salman N., Kemp A.H., Ghogho M. (2012). Low complexity joint estimation of location and path-loss exponent. IEEE Wirel. Commun. Lett..

[19] Mao G., Anderson B.D.O., Fidan B. (2007). Path loss exponent estimation for wireless sensor network localization. Comput. Netw..

[20] So H.C. (2011). Source localization: Algorithms and analysis. Handbook of Position Location: Theory, Practice and Advances.

[21] Zekavat S.A., Buehrer R.M. (2011). Handbook of Position Location: Theory, Practice and Advances.

[22] Fidan B., Çamlıca A., Güler S. (2015). Least-squares-based adaptive target localization by mobile distance measurement sensors. Int. J. Adapt. Control Signal Proc..

[23] Pahlavan K., Li X., Makela J.P. (2002). Indoor geolocation science and technology. IEEE Commun. Mag..

[24] Ioannou P.A., Fidan B. (2006). Adaptive Control Tutorial.

[25] Fidan B., Dasgupta S., Anderson B.D.O. (2013). Adaptive range measurement-based target pursuit. Int. J. Adapt. Control Signal Proc..

[26] Shames I., Dasgupta S., Fidan B., Anderson B.D.O. (2012). Circumnavigation from distance measurements under slow drift. IEEE Trans. Autom. Control.

[27] Umay I., Fidan B., Yüce M.R. Wireless capsule localization with unknown path loss coefficient and permittivity. Proceedings of the 2015 IEEE/RAS International Conference on Advanced Robotic.

[28] Whitehouse K., Karlof C., Culler D. (2007). A practical evaluation of radio signal strength for ranging-based localization. ACM SIGMOBILE Mob. Comput. Commun. Rev..

[29] Fabresse F.R., Caballero F., Ollero A. Decentralized simultaneous localization and mapping for multiple aerial vehicles using range-only sensors. Proceedings of the 2015 IEEE International Conference on Robotics and Automation.

